# The Effectiveness of a 6 Week Family Intervention in Reducing ADHD Symptoms and Promoting Emotional Regulation: A Feasibility Study

**DOI:** 10.1192/bjo.2026.11053

**Published:** 2026-06-30

**Authors:** Hayley Graves, Hannah Merdian, Sharron Smith, Kirsty Miller

**Affiliations:** University of Lincoln, Lincoln, United Kingdom

## Abstract

**Aims:**

Attention Deficit Hyperactivity Disorder (ADHD) is one of the most common neurodevelopmental disorders in children and adolescents with an estimated prevalence of up to 5% in children. Since 2019 there has been an average annual increase of 18% nationally, in ADHD medication prescriptions. Therefore with the uncertainty around the long-term effects of medication use and the side effects as we currently understand them, there is the need for other interventions to be available for children with ADHD.

This research evaluated the feasibility of a yoga and mindfulness-based family programme developed specifically for children with an ADHD diagnosis; designed to reduce ADHD behaviours, improveparent-child relationships, and emotional regulation.

**Methods:**

17 families with children aged 6–11 completed a 6-week yoga and mindfulness programme. Families completed the E SWAN assessment forms which include questionnaires for ADHD, Disruptive Mood Dysregulation Disorder, Depression, Anxiety and Panic Disorder. Families were also interviewed post intervention.

**Results:**

The quantitative findings were positive with significant outcomes: 94.1% of participants were reported to show an improvement in ADHD symptoms post intervention, with an overall 38.6% reduction in symptoms. The qualitative findings evidenced that post intervention the children were able to independently implement emotional regulation techniques, parents/carers were also able to implement strategies to regulate their own emotions, improving their ability to co-regulate their child.

**Conclusion:**

This is the first study using this particular family intervention that has been developed specifically for children with ADHD diagnosis. The results highlighted the programme was effective in reducing ADHD symptoms, improving the parent-child relationship and emotional regulation.

